# Assessing the Effect of Exogenous Melatonin on Orthodontic Tooth Movement

**DOI:** 10.7759/cureus.65885

**Published:** 2024-07-31

**Authors:** Sanjana Thiagarajan, Umarevathi Gopalakrishnan

**Affiliations:** 1 Department of Orthodontics and Dentofacial Orthopedics, Sri Venkateswara Dental College and Hospital, Chennai, IND

**Keywords:** bone deposition, alveolar bone resorption, bone metabolism, orthodontic tooth movement, melatonin

## Abstract

Objective: To examine the effect of orthodontic tooth movement on experimental Wistar rats by synthesizing melatonin formulation for administration and conducting serological analysis of alkaline phosphatase (ALP) and melatonin, along with histological evaluation and immunohistochemistry analysis of ALP and interleukin-6 (IL-6) in both control and experimental groups.

Methodology: Nine male Wistar rats were randomly divided into negative (*n* = 3), positive control (*n* = 3), and experimental groups (*n* = 3). Endogenous melatonin levels (pg/mL) were assessed, and an orthodontic force of 10 cN was applied to positive control and experimental groups using a ligature wire. The experimental group received a daily dose of 10 mg/kg melatonin via intraperitoneal injection. After eight weeks, blood samples and radiographs were collected, and mandible sections were prepared for histopathological and immunohistochemical evaluation.

Results: The radiographic evaluation shows minimal orthodontically induced tooth movement in comparison to the positive control group. In serological analysis, ALP was found to be increased in rats under the melatonin group. And, in the immunohistochemical evaluation, ALP was found to be increased in the melatonin group, whereas IL-6 was found to be decreased in the same (*P *= 0.027).

Conclusions: The study elucidates that the administration of exogenous melatonin during orthodontic tooth movement in Wistar rats induces bone formation and inhibits resorption, eventually decelerating the process of orthodontic tooth movement. Our study emphasizes melatonin’s dualistic role in stimulating bone production and suppressing resorption, offering potential therapeutic clinical implications in orthodontics.

## Introduction

Aaron Lerner extracted melatonin, also known as 5-methoxy-N-acetyl tryptamine, from the bovine pineal gland in 1958 [1]. Melatonin is primarily secreted by the pineal gland and secondarily produced in several other organs like the retina, ovary, lens, GIT, and immune cells [2-6]. Extra pineal melatonin levels have been detected in saliva, plasma, and gingival tissue [7].

Melatonin governs the human body's circadian rhythm and biological clock has a strong antioxidant action on reactive oxygen species and can help to mitigate the negative impacts of free radical damage in the body. The capability of melatonin to permeate mitochondria plays a vital role in averting free radical production, oxidative damage, and apoptosis. It effectively scavenges free radicals, stops lipid peroxidation, and neutralizes harmful reactive oxygen and nitrogen species. It also enhances the body’s antioxidant defenses by up-regulating enzymes like superoxide dismutase, catalase, and glutathione peroxidase, shielding them from oxidative harm. The antioxidant benefits of melatonin have been demonstrated in both in vivo and in vitro [8]. Melatonin also has a range of complex roles, such as osteopromotion and immunomodulation [7]. It regulates bone metabolism, enhances bone formation using the increased proliferation of preosteoblasts, and regulates systemic hormones such as parathyroid hormone (PTH), calcitonin, and scavenges free radicals produced by osteoclasts during bone resorption while also protecting bone cells against oxidative damage [5,7,9].

Since orthodontic tooth movement involves alveolar bone remodeling, any factor influencing osteoblastic or osteoclastic function can hypothetically affect orthodontic tooth movement as well. Recent studies on dental implants revealed melatonin's positive effects on bone mineral density, wound healing, and reduced pocket depth, enhancing periodontal health and implant stability [7,10,11]. Nevertheless, the role of melatonin in orthodontic tooth movement has not been addressed in the orthodontic literature so far. Hence, the current study aims to determine the role of synthetic melatonin on orthodontic tooth movement through an animal experiment.

## Materials and methods

The study, approved by the Institutional Ethics Committee at Sri Venkateswara Dental College and Hospital (IEC/SVDCH/2107) and the Institutional Animal Ethics Committee at Saveetha Dental College and Hospital (BRULAC/SDCH/SIMATS/IAEC/04-2022/100), involved nine young adult male Wistar rats (230-250 g). Female rats were excluded due to estrogen's influence on melatonin and orthodontic tooth movement [12]. Rats weighing 200-250 g were selected to endure experiment-related distress [13]. Housed at 23 +/- 2 °C with a 12-hour daylight/darkness cycle, rats had free access to food and water for standardized melatonin stimulation throughout (Table 1). 

**Table 1:** Characteristics of the animals included in the study.

Species	Strain	Total number	Sex	Weight	Supplier detail	Route of blood withdrawal	Sacrifice method
Rats	Wistar	9	Male	~250 g	Mass Biotech, Chennai	Retro orbital	CO_2_ chamber

The rats were randomly divided into three groups a positive control (rat with orthodontic force and without exogenous melatonin), a negative control (without force and exogenous melatonin), and an experimental (with orthodontic force and exogenous melatonin), with three animals in each group. The pretreatment endogenous melatonin levels were assessed using an ELISA kit (S.R. Group Chemicals, Mumbai, India). The protocol was to induce orthodontic force using an orthodontic ligature wire of 0.012" threaded, encircling the lower incisor in both the positive control and experimental groups, where the exogenous melatonin was to be administered only to the latter. The negative group receives neither the drug nor the orthodontic force (Table 2).

**Table 2:** Description of the groups.

Group	Intervention
Group I: Negative control (*n* = 3)	No orthodontic force; no melatonin
Group II: Positive control (*n* = 3)	With orthodontic force; no melatonin
Group III: Experimental (*n* = 3)	With orthodontic force and melatonin

**Figure 1:**
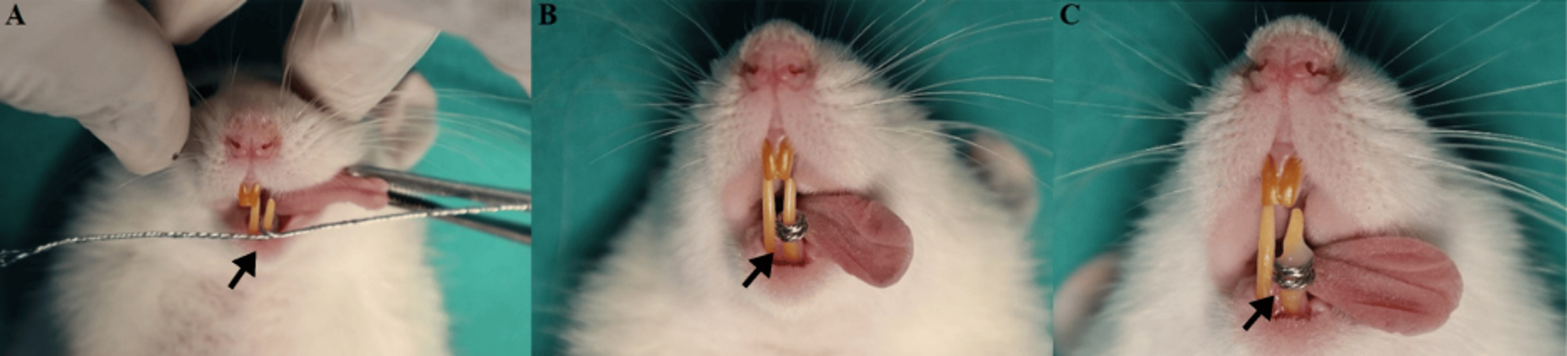
(A) Threaded ligature wires coiled around the lower incisor, with the arrow indicating the coiled wires; (B) the coil seated properly using a tucker, with the arrow indicating the seated coil; (C) the coil secured with type-1 glass ionomer cement, with the arrow indicating the luted coil.

The animals were anesthetized by an intraperitoneal injection of 10% ketamine and 2% xylazine and mounted on a jaw retraction board. Ligature wires were custom threaded and coiled around the lower incisor in a separator-like fashion, with a force of 10 cN. To prevent it from disengaging, the wire was luted with glass ionomer cement type 1 (GC-Gold Label, Luting and Lining Cement, GC Corporation, Tokyo, Japan) (Figure 1).

**Figure 2:**
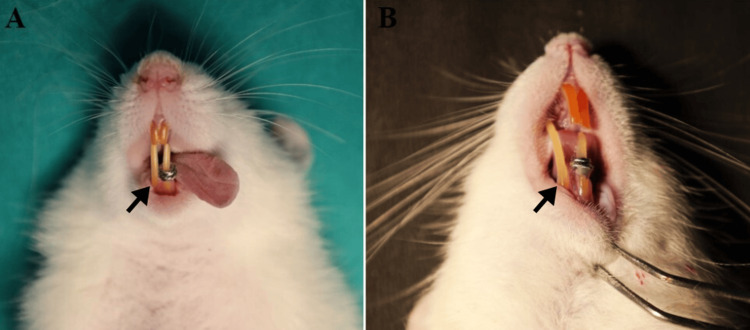
(A) Incisors before eight weeks; (B) incisors at the end of eight weeks. Arrows indicate the amount of tipping before and after eight weeks.

The experimental group received daily intraperitoneal injections of melatonin (6855623-5G; Sisco Research Laboratories, Pvt. Ltd., Mumbai, India) at a dose of 10 mg/kg body weight throughout the experimental period of eight weeks (Figure 2).

**Figure 3:**
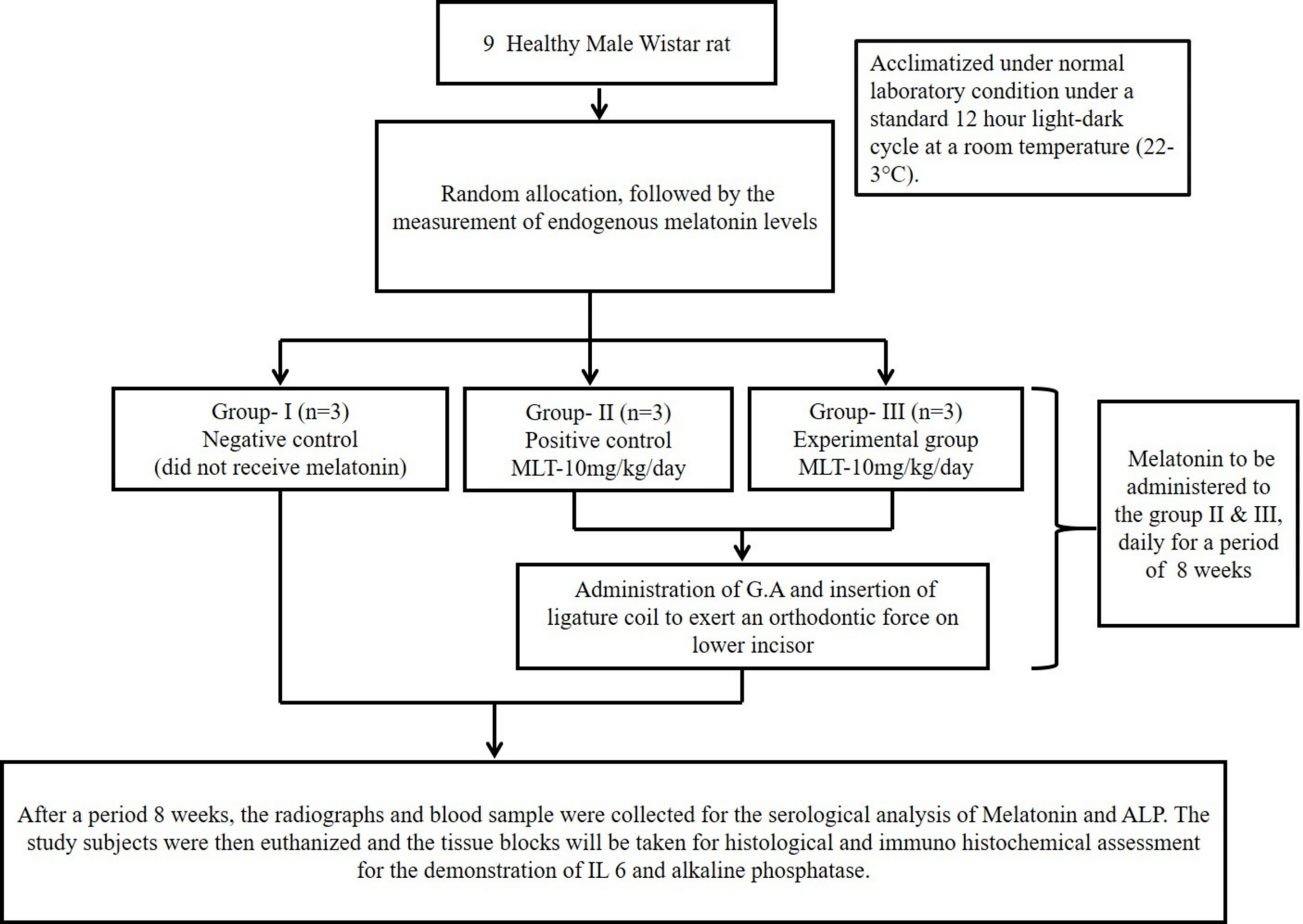
The methodology is summarized in a flowchart. Image credit: Authors' creation. MLT, melatonin; GA, general anesthesia; ALP, alkaline phosphatase

Before dissection, the rats were anesthetized and 1 mL of blood was obtained via the retro-orbital route for serum analysis after which they were sacrificed in a CO_2_ chamber. The mandibular jaws were dissected, and alveolar segments were prepared for radiographic, serological, histological, and immunohistochemical evaluation (Figure 3).

Radiographic Evaluation

The rats were anesthetized before taking the radiographs (submental-vertex), and the radiation was exposed according to the following parameters: 7 mA, 70 kVp, exposure time of 9 seconds, and at a distance of 30 cm.

Biochemical Evaluation

The acquired blood samples were left undisturbed at room temperature for 20-30 minutes and centrifuged at 2,700 rpm for 10 minutes to retrieve the resultant supernatant serum. This serum was used to determine the levels of alkaline phosphatase (Greiner Diagnostics GmbH) and melatonin.

Histopathological Evaluation

Three histological cuts along the incisors around the alveolar bone of the mandible were stained with hematoxylin and eosin after being fixed with a 10% neutral buffered formalin and decalcified with 14% ethylenediaminetetraacetic acid and embedded in paraffin. Light microscopy and an image analysis application were used to investigate the histological parameters of resorption and deposition areas.

Immunohistochemical Evaluation

After deparaffinizing and rehydrating the sections, endogenous peroxidase activity was inhibited for 20 minutes with 3% hydrogen peroxide in methanol. The sections were rinsed twice in phosphate-buffered saline (PBS) for 10 minutes. Antigenic recovery involved submerging the sections in citrate buffer (pH = 6) and heating them in a microwave oven at 700 watts (two 5-minute cycles) before rinsing them twice (10 minutes each) with PBS. Nonspecific ligations were inhibited for 45 minutes by submerging the sections in serum. Primary antibodies (anti-IL-6) were employed at a 1:100 concentration at room temperature for 24 hours. The sections were rinsed twice in PBS for 10 minutes, submerged in biotinylated serum for 30 minutes, and rinsed thoroughly more in PBS for 10 minutes before streptavidin was administered for 30 minutes. After washing, the sections were then stained using 3,3′-diaminobenzidine tetrahydrochloride hydrate. Hematoxylin was used as a counterstain. Slides were treated with alkaline phosphatase and IL-6 antibodies, and immunopositivity was determined.

## Results

Radiographic evaluation

**Figure 4:**
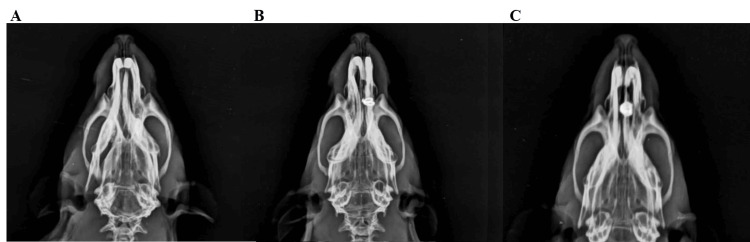
Radiographic evaluation. (A) Group I showing no signs of tooth movement; (B) Group II portraying considerable incisor tipping movement when compared with the other two groups; (C) Group III showing minimal signs of tooth movement with no major discernable tipping.

Radiographic evaluation denoted that Group II (positive control) showed an increased amount of incisor tipping movement in comparison to Group III (melatonin) (Figure 4). 

Biochemical evaluation

The serum from the collected blood samples was used to measure the levels of melatonin and alkaline phosphatase (ALP) using their respective ELISA kit. Statistical analysis was performed on the results of the serological evaluation of melatonin and ALP of all the groups and found a significant difference among the three (*P *= 0.027) (Tables 3-5).

**Table 3:** Serum melatonin (conc. pg/mL)

Groups	Pretreatment			After eight weeks		
	Sample 1	Sample 2	Sample 3	Sample 1	Sample 2	Sample 3
Group I: Negative control	137.031	140.101	138.153	137.336	140.121	138.032
Group II: Positive control	142.056	137.354	153.056	142.457	136.189	154.346
Group III: Experimental group	140.771	139.032	136.342	151.336	147.342	146.852

**Table 4:** Serum alkaline phosphatase level in rats (after eight weeks).

S. no.	Group	Sample 1	Sample 2	Sample 3
1	Group I (Negative control)	138.123	135.06	137.121
2	Group II (Positive control)	182.744	180.093	179.126
3	Group III (Experimental)	187.972	189.158	187.129

**Table 5:** Parametric data from each group. SD, standard deviation

Standardized achievement percentile (SAP)	N	Mean	Mean ± SD	*P*-value
1.00	3	136.7680	1.56171	0.027
2.00	3	180.6543	1.87318	
3.00	3	188.0863	1.01932	

However, the Bonferroni test revealed a notable difference between the positive control (Group II) and experimental melatonin group (Group III) at the level of *P* = 0.05, indicating a positive increase in ALP in Group III (Table 6).

**Table 6:** Statistical and multiple comparisons using the Bonferroni test.

VAR00001 (I)	VAR00001 (J)	Mean difference (I-J)	Std. error	Sig.	95% confidence interval	
					Lower bound	Upper bound
1.00	2.00	-43.88633^*^	1.24604	.000	-47.9826	-39.7900
	3.00	-51.31833^*^	1.24604	.000	-55.4146	-47.2220
2.00	1.00	43.88633^*^	1.24604	.000	39.7900	47.9826
	3.00	-7.43200^*^	1.24604	.003	-11.5283	-3.3357
3.00	1.00	51.31833^*^	1.24604	.000	47.2220	55.4146
	2.00	7.43200^*^	1.24604	.003	3.3357	11.5283

Histological evaluation

**Figure 5:**
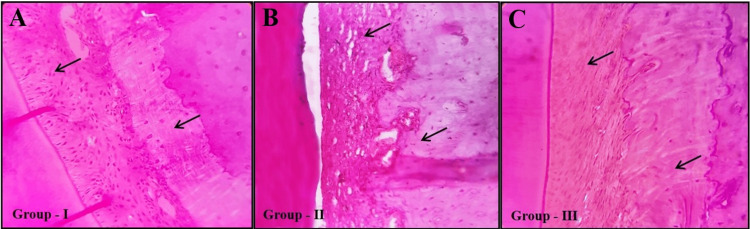
Histopathological evaluation demonstrating the tooth tissue with adjoining mandibular alveolar bone in control (A) and experimental groups (B and C). Osteogenic alterations depicting increased cortical bone thickness and periodontal ligament due to experimental orthodontic force/tooth movement are observed in both Group II (orthodontic force without melatonin) and Group III (orthodontic force with melatonin). However, Group II showed greater alveolar bone resorption and more cortical bone cellular changes than Group III. Asterisk indicates dentin; arrows, cortical bone; hematoxylin and eosin stain (magnification: x40).

On histological evaluation, the samples stained with hematoxylin and eosin (H&E) demonstrated an increased cortical bone thickness and periodontal ligament due to experimental orthodontic force/tooth movement in both Group II (orthodontic force without melatonin) and Group III (orthodontic force with melatonin). However, Group II showed a higher level of alveolar bone resorption with a substantially increased expression of Howship’s lacunae (resorption foci) and osteoclasts than Group III (Figure 5).

Immunohistochemical Evaluation

**Figure 6:**
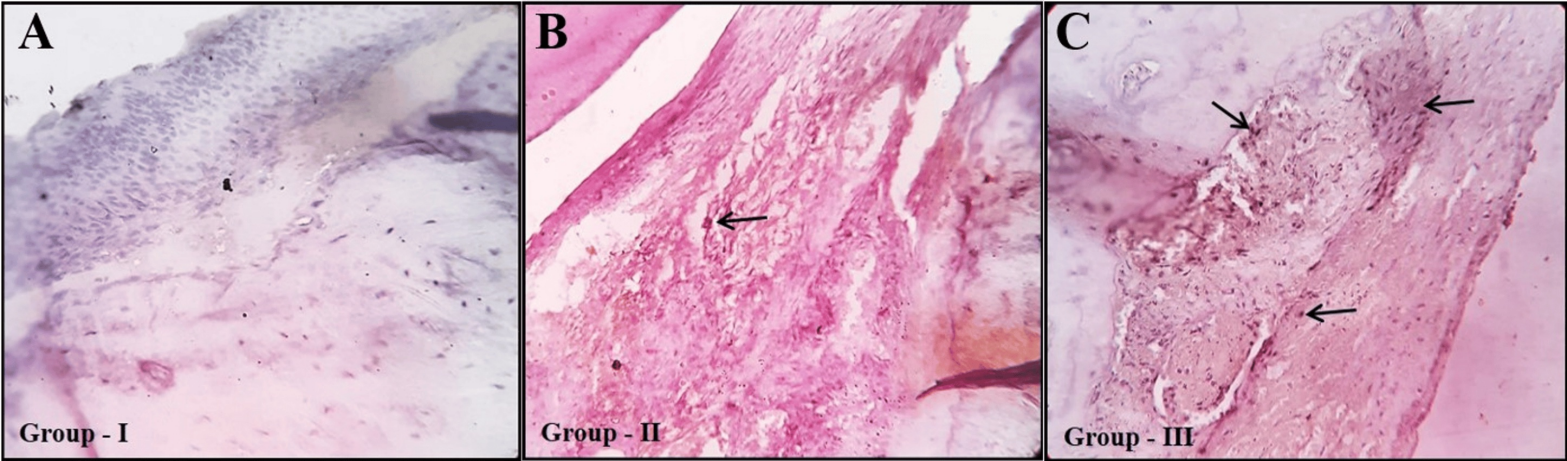
Immunohistochemical evaluation demonstrating the ALP protein expression in control (A) and experimental groups (B and C). Osteogenic alterations depicting increased positive ALP protein expression (arrows) in Group III (orthodontic force with melatonin) than Group II (orthodontic force without melatonin) when compared to the control group. IHC of ALP (magnification: x40). IHC, immunohistochemistry; ALP, alkaline phosphatase

Immunohistochemical analysis of the samples showed a positive increase in ALP protein in Group III than in Group II when compared to the control group (Figure 6).

**Figure 7:**
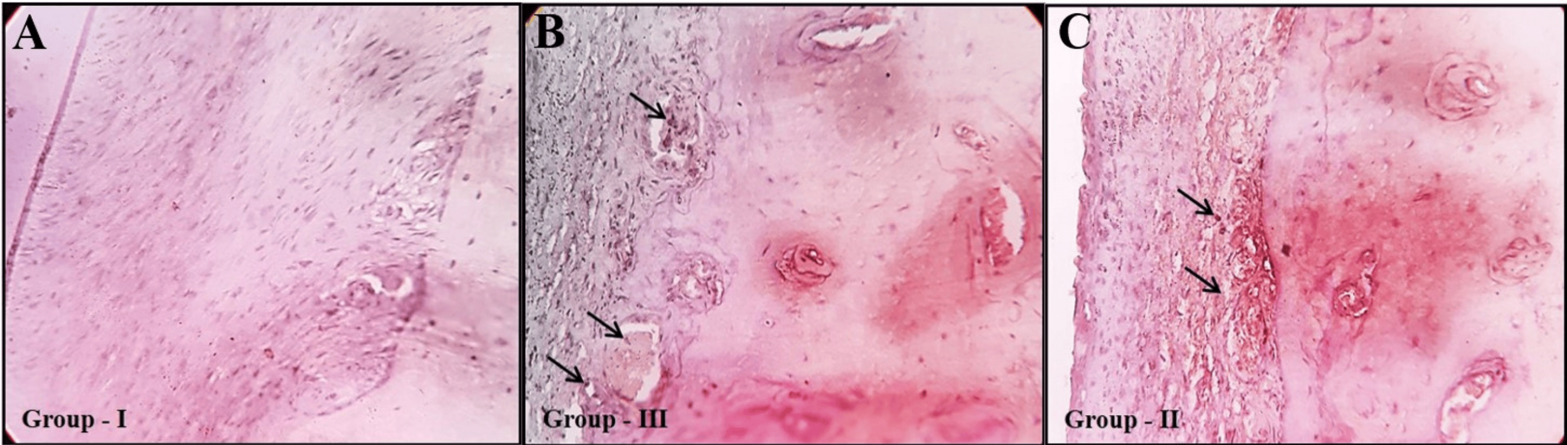
Immunohistochemical evaluation demonstrating the IL-6 inflammatory biomarker expression in control (A) and experimental groups (B and C). Osteogenic alterations depicting increased positive IL-6 inflammatory biomarker expressions (arrows) in Group II (orthodontic force without melatonin) than Group III (orthodontic force with melatonin) when compared to the control group. IHC of IL-6 (magnification: x40). IHC, immunohistochemistry; IL-6, Interleukin-6

On analyzing the inflammatory biomarker IL-6, there was an intense immune reaction for IL-6 in Group II than in Group III, when compared among the groups (Figure 7).

## Discussion

Orthodontic tooth movement is akin to a separate field of science which has in numerous scientific literature. So many intrinsic molecular details are being continuously explored. Intensive studies are available wherein every possible agent has been researched about their ability to affect the rate of orthodontic tooth movements. Caffeine, nicotine, prostaglandin, thyroxin, platelet-rich plasma (PRP), PTH, vitamin D, corticosteroid hormones, and other agents have been studied for their ability to accelerate tooth movement, whereas estrogen, triptolide, bisphosphonates, dietary calcium, and other agents have been shown to slow it down [12,14-20]. The exploration of newer molecules that can influence the rate of movement is continuously ongoing. Any agent that has a role in the turnover and remodeling of bone tissue can be tested for their influence on orthodontic tooth movement. One such agent in the recent past which has been shown to have a potential role in bone remodeling is melatonin.

Thus, the current study focuses on assessing the effect of exogenous melatonin on orthodontic tooth movement through biochemical, histological, and immunohistochemical methods in a Wistar rat model [13,21]. Female rats were excluded due to the influence of estrogen on bone turnover [12,22]. The specific weight of 250g was selected as it was known to sustain the pain, drug tolerance, and distress incurred during the study [13]. For biochemical evaluation, melatonin and ALP levels were evaluated using their corresponding ELISA kit. Previous research highlights ALP's early development presence on cell surfaces and matrix vesicles in tissues, bones, and calcifying cartilages. In the periodontium, ALP influences bone homeostasis, ligament turnover, and cementum formation [23,24]. As a result, ALP is widely used as a biomarker for bone metabolism and remodeling.

Therefore, ALP levels were compared among the groups. The results obtained from the serological test were statistically analyzed and found that the levels of ALP were increased in Group III, which was administered with melatonin when compared with the other two groups. An increase in ALP levels reflects the significant osteoblastic activity of bone deposition and mineralization by elevating inorganic phosphate levels and lowering extracellular pyrophosphate concentration, an inhibitor of mineral formation [23]. This is by the findings of the current study, which imply that melatonin may spur osteoblastic activity and promote bone deposition and mineralization.

Histological analysis revealed increased Howship's lacunae and osteoclasts in Group II, while Group III exhibited minimal resorption. Previous research observed an increase in the number of resorption lacunae, capillaries, and osteoclasts in groups with increased orthodontic tooth movement. Melatonin inhibits bone resorption by downregulating RANKL-mediated osteoclast formation and activation while also stimulating bone cell proliferation and type I collagen synthesis, according to previous research [15,19,20].

The current study's histological findings are consistent with the previous study's findings, indicating that resorption was significantly reduced in the melatonin-treated group [5,9,11]. Studies have reported that the inflammatory biomarker IL-6 has been linked to altering skeletal homeostasis and osteoclast differentiation. This cytokine produced primarily by monocytes binds to pre-osteoclast receptors and increases osteoclastogenesis, hence boosting bone resorption [25]. IL-6 also influences bone remodeling by activating JAK/STAT3 pathways in osteoblasts, leading to the release of pro-osteoclast mediators like RANKL, PGE2, IL-1, and PTHrP [26]. 

In addition, IL-6 inhibits osteoblast differentiation, disrupting the balance of healthy bone turnover. Both IL-6 and sIL-6R reduce osteoblastic differentiation by suppressing key genes like ALP, osteocalcin, and RUNX2. Furthermore, IL-6/sIL-6R signaling impairs osteoblast mineralization potential [25-28]. Altogether, IL-6 has been demonstrated to have both pro-osteoclastic and anti-osteogenic effects, resulting in net resorption of bone [25]. Thus, in the current study, IL-6 was used as an inflammatory biomarker in immunohistochemical analysis. According to immunohistochemical analysis, ALP expression was higher in Group III, while IL-6, the inflammatory biomarker known to promote osteoclastogenesis and stimulate bone resorption, was relatively low.

On cumulating the results from the current study and comparing with the existing literature on melatonin of its effect on bone architecture and metabolism, not only does melatonin promote bone formation but also inhibits resorption. This may suggest that the duration of orthodontic therapy might be increased as orthodontic tooth movement requires a balance in remodeling with resorptive and depository patterns. 

Limitations

While the findings suggest a potential positive effect of melatonin on both bone formation and reduced resorption, additional research is necessary to fully elucidate the specific molecular mechanisms involved and to evaluate the long-term consequences. The study is limited by a small sample size and the inclusion of only female rats. Additionally, due to practical constraints, we were unable to band the molar or use it as an anchorage for incisor tooth movement. Future studies should employ improved methodologies to address these limitations.

## Conclusions

The findings of our study indicate the potential possibility of melatonin having a positive role in augmenting bone deposition and decreasing bone resorption. The findings can be extrapolated clinically in situations that demand an increased bone formation like areas of alveolar bone around the anchor teeth, areas of bone around mini-implants, pins used in distraction osteogenesis, etc. It can also be used in patients who are prone to increased bone resorption during orthodontic treatment presenting a relative contraindication to tooth movement like diabetes mellitus, pregnancy and lactating mothers, adult patients, periodontally compromised individuals, individuals with osteoporosis, etc., can be handled by the local administration of melatonin.
